# GABPB1 plays a cancer-promoting role in non-small cell lung cancer

**DOI:** 10.1007/s12672-024-00914-4

**Published:** 2024-03-11

**Authors:** Tuo Wang, Cong Cao, Yu Fan, Jialing Xu, Tao Hua, Jie Ding, Zejie Liu, Beili Wang, Juanwen Lian

**Affiliations:** Department of Oncology, Xi’an Chest Hospital, Xi’an, 710100 Shaanxi China

**Keywords:** NSCLC, GABPB1, Tumor immune microenvironment (TIME), Cell proliferation, Apoptosis

## Abstract

**Background:**

GABPB1, the gene that encodes two isoforms of the beta subunit of GABP, has been identified as an oncogene in multiple malignant tumors. However, the role and mode of action of GABPB1 in malignant tumors, especially in lung cancer, are not well understood and need further research.

**Methods:**

Our research focused on examining the biological function of GABPB1 in NSCLC (Non-Small Cell Lung Cancer). We analysed tumor data from public databases to assess the expression of GABPB1 in NSCLC  and its correlation with patient prognosis and investigated GABPB1 expression and methylation patterns in relation to the tumor microenvironment. In parallel, experiments were conducted using short hairpin RNA (shRNA) to suppress the GABPB1 gene in human lung cancer cells to evaluate the effects on cell proliferation, viability, and apoptosis.

**Results:**

GABPB1 was widely expressed in various tissues of the human body. Compared to that in normal tissues, the expression of this gene was different in multiple tumor tissues. GABPB1 was highly expressed in lung cancer tissues and cell lines. Its expression was associated with molecular subtype and cellular signalling pathways, and a high level of GABPB1 expression was related to a poor prognosis in lung adenocarcinoma patients. The expression and methylation of GABPB1 affect the tumor microenvironment. After suppressing the expression of GABPB1 in both A549 and H1299 cells, we found a decrease in cell growth and expression, the formation of clones and an increase in the apoptosis rate.

**Conclusions:**

Our research verified that GABPB1 promotes the tumorigenesis of NSCLC and has an inhibitory effect on tumor immunity. The specific role of GABPB1 may vary among different pathological types of NSCLC. This molecule can serve as a prognostic indicator for lung adenocarcinoma, and its methylation may represent a potential breakthrough in treatment by altering the tumor immune microenvironment in lung squamous cell carcinoma. The role and mechanism of action of GABPB1 in NSCLC should be further explored.

## Introduction

Over 80% of all lung cancer cases are attributed to non-small cell lung cancer (NSCLC) [1]. Although platinum-based chemotherapy has been a mainstay treatment option for NSCLC for the past two decades, its impact on overall survival has been limited [2]. With the emergence of comprehensive genomics, we have identified target drugs, such as kinase inhibitors of oncogenic receptor tyrosine kinases (RTKs), including EGFR, ALK, MET, and ROS-1, as well as downstream target kinases, such as BRAF [3]. However, this approach benefits only certain patients and is limited to tumors that rely solely on the oncogenic activity of a single oncogene [4, 5]. Therefore, novel biomarkers and molecular targets that impact therapeutic strategies and the progression of NSCLC need to be identified. Given these challenges, it is imperative to focus on identifying novel biomarkers and molecular targets that can positively impact therapeutic strategies and NSCLC progression. Numerous studies have focused on the role of transcription factors that act as oncogenes or tumor suppressors in the occurrence and development of lung cancer [6, 7]. These transcription factors can serve as sensitive diagnostic markers or promising prognostic markers [8]. GA-binding protein (GABP) is a nuclear-encoded E-twenty-six (ETS) transcription factor that consists of the DNA-binding protein GABPa and the transactivated GABPb subunit [9]. This molecule has been found to play a role in the progression of multiple cancers, including chronic myeloid leukaemia, liver cancers, and glioblastomas [10–12]. GABPB1, a gene that encodes two different isoforms of the beta subunit of GABP (GABPβ1L and GABPβ1S), may play a role in regulating the interaction between mitochondria and the nucleoplasm and maintaining the balance between the energy metabolism of cells and the ability to resist oxidative damage [13]. However, the relationship between GABPB1 and malignant tumors, especially lung cancer, has largely not been determined. In this study, we utilized database information to observe the expression of GABPB1 in lung cancer and its relationship with clinical characteristics and prognosis, as well as its impact on tumor immunity. Additionally, we used human cell lines to investigate the effects of knocking out GABPB1 on normal cellular functions, further exploring its role in NSCLC.

## Materials and methods

### Tissue samples

A total of 15 paired NSCLC tissues and normal lung tissues were collected after resection from Xi'an Chest Hospital between December 2020 and January 2022. A total of 9 males and 6 females were included, and the mean age was 56 years (range, 38–71 years). Eight of these patients had squamous cell carcinoma, and 7 had adenocarcinoma. The distance between normal tissue and lung cancer tissue was more than 3 cm. The frozen tissues were maintained at -80 degrees Celsius. Before surgery, the patients did not undergo any treatment. This study was approved by the Ethics Committee of Xi'an Chest Hospital (no. s2020-0029).

### Cell culture

Human NSCLC cell lines were purchased from the Cell Bank of Type Culture Collection of the Chinese Academy of Sciences. The cells were cultured at 37degrees Celsius with 5% CO_2_ in a humidified incubator. NCI-H1299, NCI-H1975 and 95-D cells were cultured in DMEM (Gibco) supplemented with 10% FBS (Gibco) and 1% penicillin‒streptomycin (HyClone), and A549 cells were maintained in DMEM/F12 medium (Sigma‒Aldrich; Merck KGaA) supplemented with 10% FBS (Gibco; Thermo Fisher Scientific, Inc.) and 1% penicillin‒streptomycin (HyClone; GE Healthcare).

### Tissue immunohistochemistry

Four micron sections were dried at 60 degrees Celsius overnight. The sections were deparaffinized, rehydrated and subjected to antigen retrieval according to established methods. Endogenous peroxidase was blocked with 3% hydrogen peroxide at room temperature for 10 min, washed with PBS for 3 min three times, blocked at room temperature for 30 min with normal goat serum (Abcam), and incubated with a rabbit anti-GABPB1 primary antibody (1:400; cat. no. HPA019653; Atlas Antibodies) for 90 min at room temperature. After washing, goat anti-rabbit IgG antibody (1:1000; cat. no. Ab6721; Abcam) was subsequently added, after which the sections were incubated at room temperature for 1 h and washed with PBS. Then, 3′,3′-diaminobenzidine (Beyotime Institute of Biotechnology) was used to develop the colour for 3 min at room temperature. The slides were counterstained with haematoxylin for 5 min at room temperature, mounted after rinsing with water, and observed using light microscopy (Olympus Corporation).

### Lentiviral vectors and cell transfection

For analysis of the role of GABPB1 in NSCLC, shGABPB1 was constructed with recombinant lentivirus and transfected into the A549 and H1299 cell lines. We examined the efficiency of the transfection using a fluorescence microscope. The cells transfected with shGABPB1 or shCtrl were defined as the experimental group and negative control group, respectively. Lentiviruses containing the RNA interference sequence of the target gene GABPB1 and the negative control were chemically synthesized by Shanghai Genechem (Shanghai Genechem Co., Ltd., Shanghai, China). The sequences of GABPB1 shRNA and negative control shRNA were 5ʹ-AGAACCAAATCAACACAAA-3ʹ and 5ʹ-TTCTCCGAACGTGTCACGT-3ʹ, respectively. Cell transfection was carried out according to the manufacturer’s instructions for NSCLC cell lines. NSCLC cells were cultivated in 6-well plates, and negative control lentivirus or the GABPB1-shRNA lentivirus was added according to the multiplicity of transfection. Two groups of cells were collected to determine the knockdown efficiency using a fluorescence microscope (MicroPublisher 3.3, Olympus) after 72 h of transfection. RNA was extracted using SuperfecTRI (Shanghai Pufei Biotechnology Co., Ltd.) according to the manufacturer’s protocol. An ultraviolet spectrophotometer was used to measure the concentration of the extracted RNA. A total of 5 µg of RNA was reverse transcribed using an M-MLV reverse transcriptase kit (Promega Corporation) according to the manufacturer’s protocol. The forward and reverse primers (Shanghai Genechem Co., Ltd.) used were as follows: GABPB1, 5ʹ -CCTAACAGATGAAACGGGTGT-3ʹ and 5ʹ-CCACTGGTTGGAATAGAGTGC-3ʹ; and GAPDH, 5ʹ -TGACTTCAACAGCGACACCCA-3ʹ and 5ʹ-CACCCTGTTGCTGTAGCCAAA-3ʹ. We used a SYBR Master Mixture Real-Time PCR System (TaKaRa Biotechnology Co., Ltd., Dalian, China) to perform RT‒PCR in 12 µl reactions under the following reaction conditions. GAPDH was used as the internal control. RT‒PCR was performed according to the following conditions. Predenaturation: 30 s at 95 degrees Celsius. Denaturation: 40 cycles of 5 s at 95 degrees Celsius and 30 s at 60 degrees Celsius. Dissociation: 15 s at 95 degrees Celsius, 30 s at 60 degrees Celsius, and 15 s at 95 degrees Celsius. The relative gene expression levels were measured and compared via the 2 − ΔΔCT analysis program.

### Colony formation assay

shGABPB1-transfected and shCtrl-transfected cells were digested with trypsin (Sangon Biotech Co., Ltd., Shanghai, China) when they were in the logarithmic growth stage. The cells in each group were inoculated in a 6-well plate culture plate according to the growth of the cells (400–1000 cells/well), and three replicate wells were used. The cell clones were photographed under a fluorescence microscope when the inoculated cells were cultured for 14 days or the number of cells in most single clones was more than 50, 4% paraformaldehyde (Sinopharm Chemical Reagent Co., Ltd., Shanghai, China) was added in each pore, the cells were fixed for 30–60 min and washed by PBS, and clean and impurity-free crystal violet dye (Sangon Biotech Co., Ltd., Shanghai, China) solution was added to each pore for 20 min. Then, the samples were washed twice with phosphate-buffered saline (PBS), and colonies were counted manually.

### 3-(4,5-Dimethylthiazol-2-Yl)-2,5-diphenyltetrazolium bromide (MTT) assay

For measurement of cell viability, two groups of transfected cells were seeded into a 96-well culture plate at a density of 2000 cells/well and allowed to grow to subconfluence. MTT incubation and absorbance were performed following the manufacturer’s instructions. MTT (0.5 mg/mL; Genview PTY, Ltd., VIC, Australia) was added to each well and incubated for 4 h at 37 degrees Celsius, after which dimethyl sulfoxide (DMSO) (ShiYi Pharmaceutical Group, Shanghai, PR China) (100 µl) was added. After 10 min of shaking, cell proliferation was measured at OD490 using a microplate reader (Tecan, Durham, NC, USA). We examined the cells for five consecutive days and used the average count of three repetitions in each group as the result.

### Cell proliferation assay

shGABPB1-transfected and shCtrl-transfected cells were cultured and digested with trypsin (Sangon Biotech Co., Ltd., Shanghai, China). Then, the resuspended cells were seeded in a 96-well plate at a density of 2,000 cells/100 μl/well and incubated overnight at 37 degrees Celsius in 5% CO_2_. Green fluorescent protein (GFP), a marker of lentivirus-transfected cells, was expressed in every cell. We counted each cell at different time points with a Celigo image cytometer (Nexcelom Bioscience, Lawrence, MA, USA) and subsequently plotted the cell proliferation curve.

### Cell apoptosis assay

In this study, cell apoptosis was analysed using an Annexin V-FITC Apoptosis Detection Kit (BD Biosciences, C1063). The two groups of passaged cells were collected after transfection. When the cell confluence reached 85%, the cells were digested with 0.25% trypsin (without EDTA) and centrifuged. The cell precipitate was washed with 4 degrees Celsius prechilled D-Hanks solution (pH 7.2–7.4) and phosphate-buffered saline (PBS). The mixture was incubated with 100 μl of the cell suspension, 5 μl of Annexin V/FITC was added at room temperature for 5 min in the dark, 10 µl of 20 µg/ml propidium iodide solution (PI) and 400 µl of PBS were added, and flow detection was immediately performed. Flow cytometric analysis was performed on a Fluorescence Activated Cell Sorting (FACS) Calibur platform (Millipore Accuri™ C6 Plus; BD Biosciences).

### Western blotting

The transfected cell samples were washed twice with cold PBS and lysed in lysis buffer (1 M Tris–HCl (pH 6.8), 2% mercaptoethanol, 20% glycerol, and 4% SDS) on ice for 15 min. The protein concentration was determined with a BCA Protein Assay Kit (Beyotime Institute of Biotechnology, Shanghai, China). Equal volumes of cellular protein were subjected to 10% SDS‒PAGE (Bio-Rad Laboratories, Inc., Hercules, CA, USA) and electrotransferred to a PVDF membrane (EMD Millipore, Billerica, MA, USA). Then, the PVDF membrane was blocked using TBST solution buffer with 5% nonfat milk and incubated overnight at 4 °C with the following antibodies: rabbit anti-GABPB1 (1:2500; cat. no. HPA019653; Atlas antibodies); and rabbit anti-GAPDH (1:2500; cat. no. ab9485; Abcam). Then, goat anti-rabbit IgG antibody (1:1,0000; cat. no. Ab6721; Abcam) conjugated to horseradish peroxidase was used for 1.5 h. Finally, the immunoreactive bands were visualized using a Pierce™ ECL Western Blotting Substrate Kit (cat. no. K820; BioVision, Inc.) and digitized with Quantity One software (Bio-Rad Laboratories, Hercules, CA, USA).

### Relevant database analysis

We used the Human Protein Atlas (HPA) [14] and Gene Expression Profiling Interactive Analysis (GEPIA2) [15] databases to analyse the expression of GABPB1 at the protein and RNA levels in human tissues, particularly lung and lung cancer tissues. Using the GSCA (Gene Set Context Analysis) [16] database, we analysed four aspects of NSCLC: the correlation between GABPB1 expression and tumor stage and related pathways, its prognostic value for patients, and the impact of GABPB1 and its methylation on the tumor immune microenvironment.

### Statistical analysis

The statistical analysis was performed with the SPSS 19.0 program (IBM Corporation, Armonk, NY, USA). In this study, the data collected are presented as the mean ± standard deviation (SD). Differences between two groups were determined with Student’s t test. P < 0.05 was considered to indicate statistical significance.

## Results

### GABPB1 was expressed in most normal and tumor tissues in the human body

**Fig. 1:**
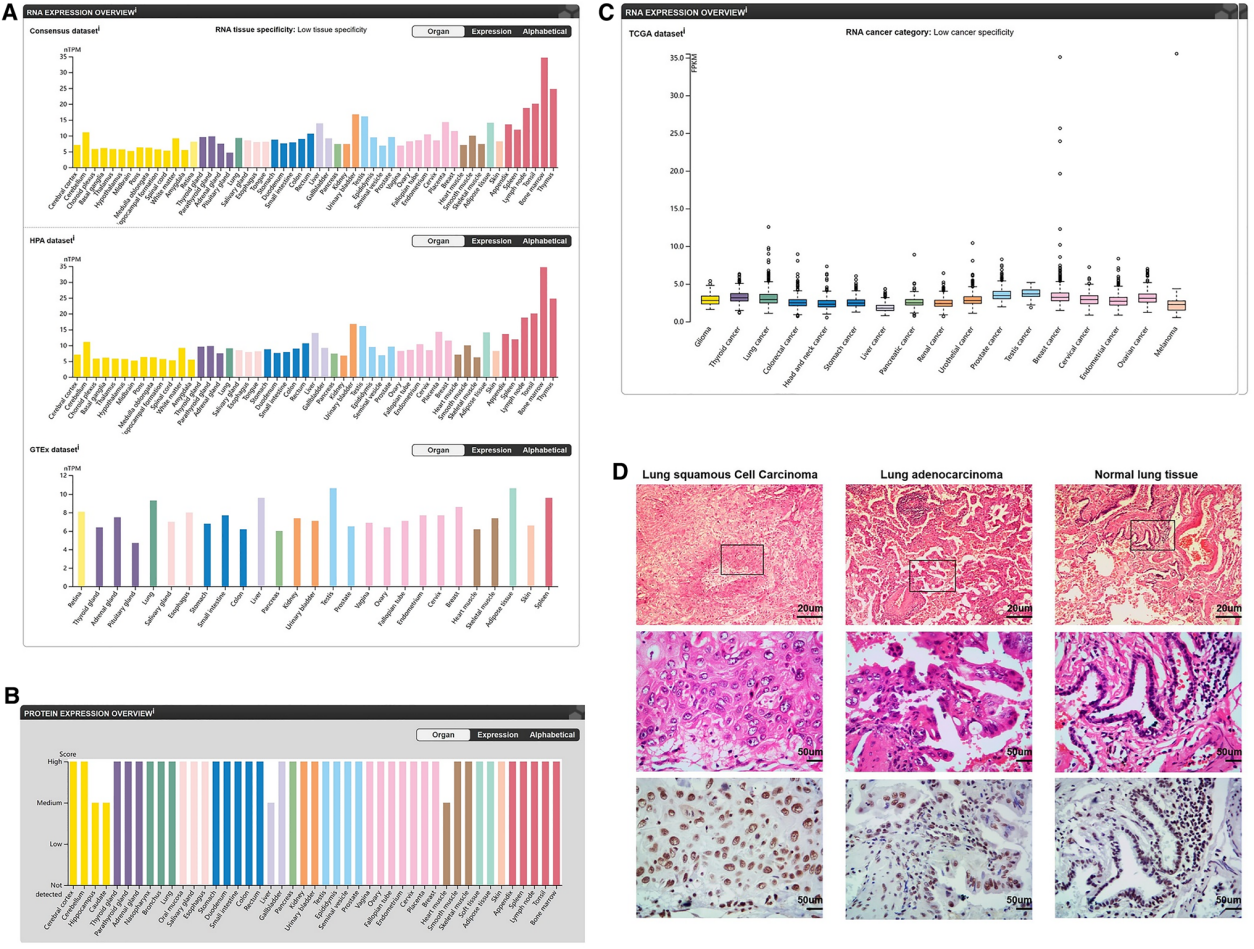
GABPB1 RNA and protein expression levels in various human tissues. **A**, **B** GABPB1 was expressed in various human tissues at both RNA and protein levels. **C** The expression of GABPB1 in normal lung and lung cancer tissues. **D** The expression of GABPB1 in postoperative lung cancer and surrounding normal tissues

After analysing the HPA database, we found that GABPB1 was expressed in various human tissues at both the RNA and protein levels, as shown in Fig. 1A and B. Human tumor data obtained from the TCGA database could be distinguished based on the protein expression of GABPB1 (Fig. 1C). We tested samples from lung cancer patients who underwent standard surgery, and the results showed that GABPB1 was expressed in alveolar epithelial cells, vascular endothelial cells, and lung cancer cells and was localized in both the nucleoplasm and cytoplasmic bodies (Fig. 1D).

### GABPB1 exhibited high expression and was associated with specific tumur characteristics in NSCLC

**Fig. 2:**
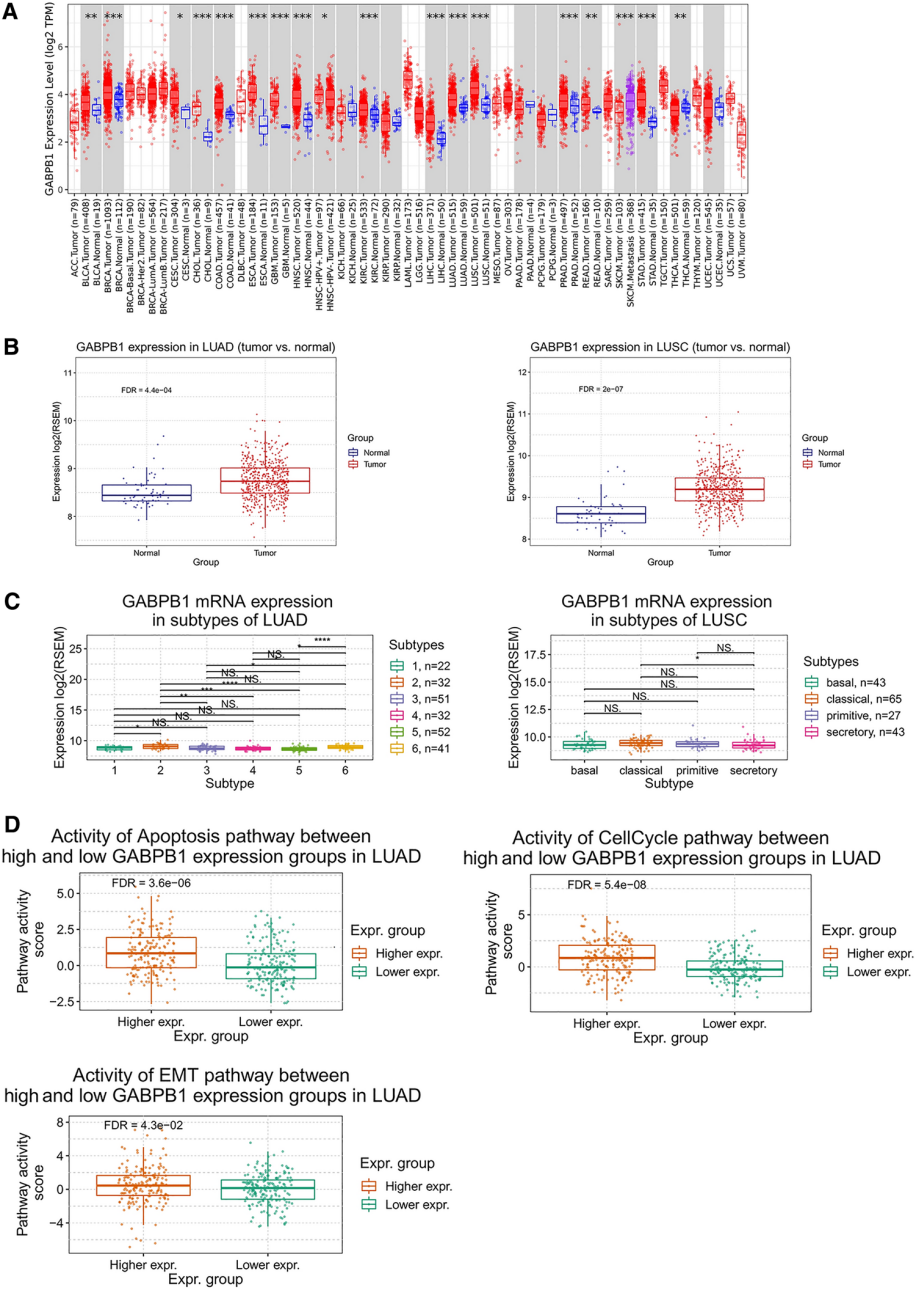
The correlation between GABPB1 and clinical pathological characteristics. **A** GABPB1 was a differential expression in tumor tissue compared with normal tissue for most tumor species. **B** GABPB1 was over-expressed in both lung adenocarcinoma and lung squamous cell carcinoma. **C** The expression of GABPB1 in lung adenocarcinoma was significantly associated with the molecular subtypes. **D** The expression of GABPB1 in lung adenocarcinoma was significantly associated with cellular signaling pathways. *P < 0.05, **P < 0.01, ***P < 0.001, ****P < 0.001

Analysis of the extensive TCGA and GTEx datasets through the use of GEPIA2 and GSCA revealed that GABPB1 was differentially expressed in tumor tissue compared with normal tissue across the majority of tumor species (Fig. 2A). Overexpression of GABPB1 was observed in both lung adenocarcinoma and lung squamous cell carcinoma (Fig. 2B). Notably, in adenocarcinoma patients, the expression of GABPB1 was also significantly associated with molecular subtypes (Fig. 2C) and tumor pathway activation, such as apoptosis, the cell cycle and EMT (Fig. 2D).

### GABPB1 was highly expressed in NSCLC cell lines

**Fig. 3:**
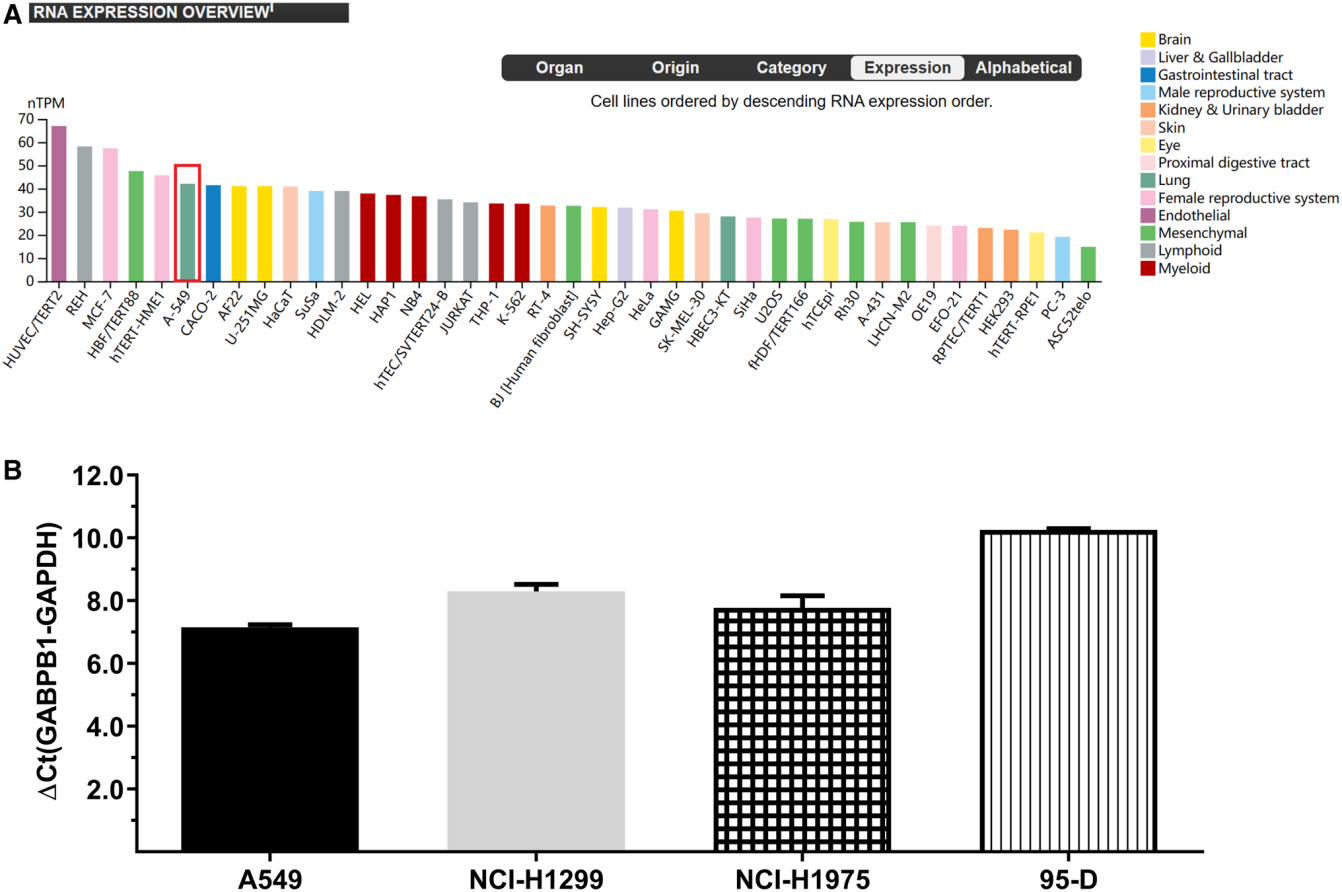
Expression of GABPB1 in four non-small-cell lung cancer cell lines

The results from the database analysis revealed that GABPB1 is expressed in multiple cell lines, with relatively higher expression in A549 cells (Fig. 3A) [14]. A comparative RT‒PCR analysis was also conducted to evaluate GABPB1 mRNA expression in other NSCLC cell lines, including H1299, H1975 and 95-D. The results showed that the expression of GABPB1 mRNA was basically equivalent in A549 and H1299 cells. Compared to that in the A549 cells obtained from the database, the expression of GABPB in H1299 cells was also relatively high. For subsequent cell function studies, we selected the A549 and H1299 cell lines.

### Knockdown efficiency of GABPB1 in non-small cell lung cancer cell lines

**Fig. 4:**
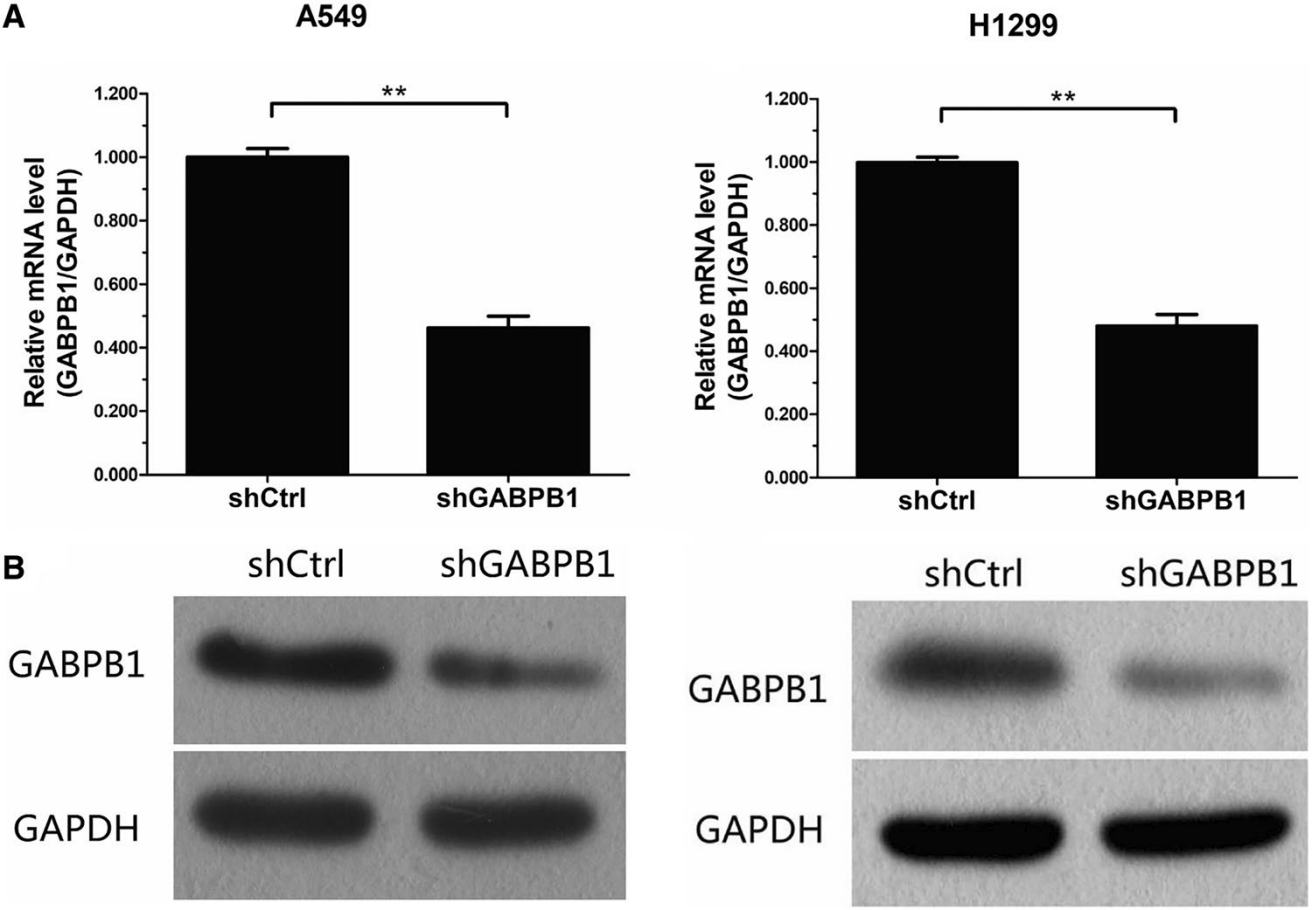
Konckdown efficiency of GABPB1 in non-small-cell lung cancer cell lines. **P < 0.01 compared shGABPB1 group with shCtrl group

A549 and H1299 cell lines were infected with LVpGCSIL-004PSC51554-1 (GABPB1-shRNA lentivirus) and psc3741 (shCtrl lentivirus). After 3 days of transfection, we observed that the percentage of infected cells was > 80%, and the cell morphology remained normal in both experimental groups. The cells continued to grow normally after transfection. The RT–PCR results indicated a significant decrease in GABPB1 mRNA expression in A549 or H1299 cells in the experimental group compared to that in the negative control group (Fig. 4A), P < 0.05. Additionally, Western blotting demonstrated a decrease in GABPB1 protein expression in the experimental group, as demonstrated in Fig. 4B, for both cell lines. Lentivirus transfection effectively downregulated the expression of GABPB1 without negatively affecting the normal growth of cells.

**Fig. 5:**
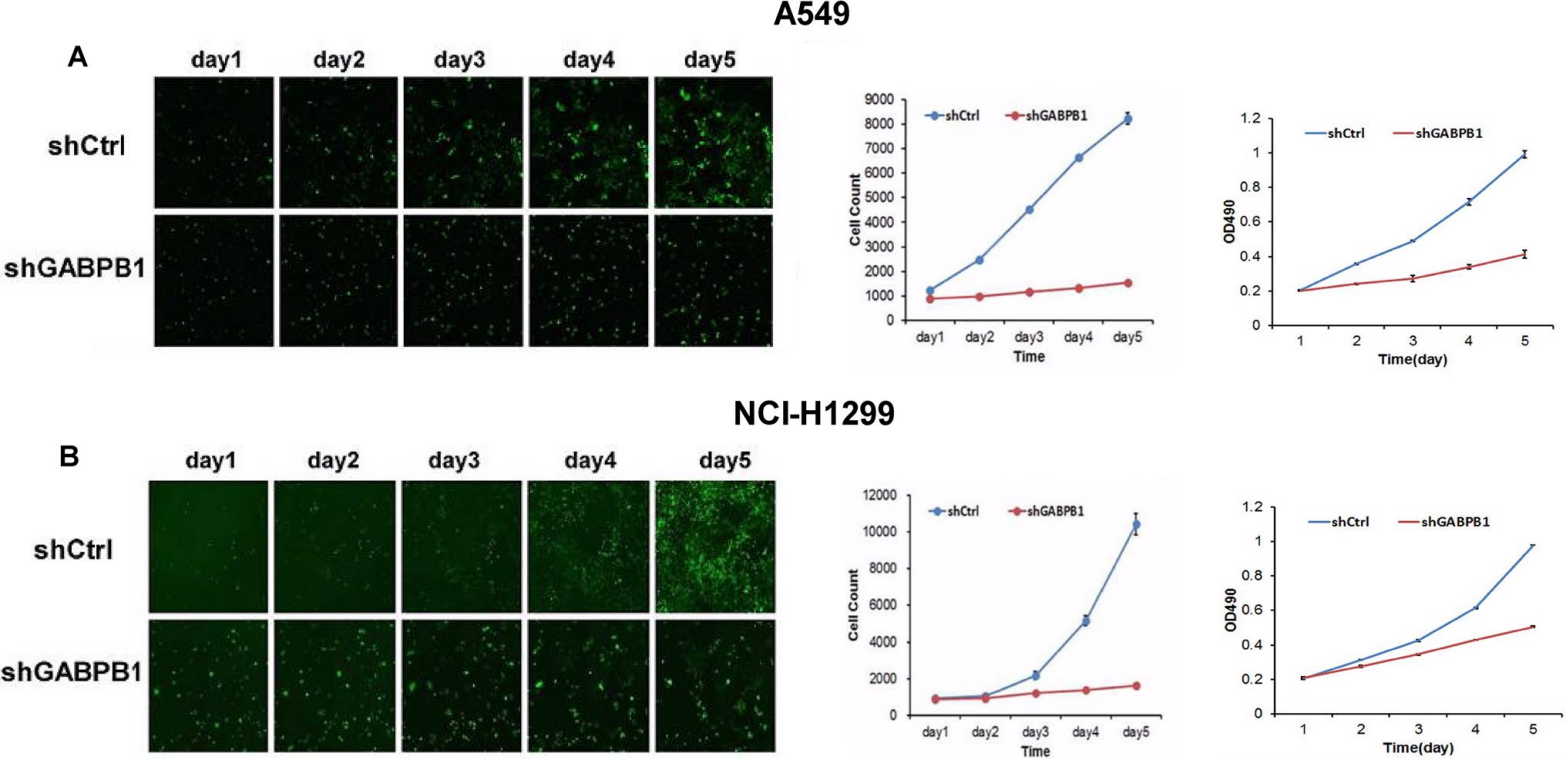
The cell growth and proliferation ability determined by Celigo assay and MTT. **A** The cell growth and proliferation of shGABPB1 group decreased compared with shCtrl group in A549 cells. **B** The cell growth and proliferation of shGABPB1 group decreased compared with shCtrl group in H1299 cells

### Knockdown of GABPB1 inhibited cell growth and proliferation

**Fig.6:**
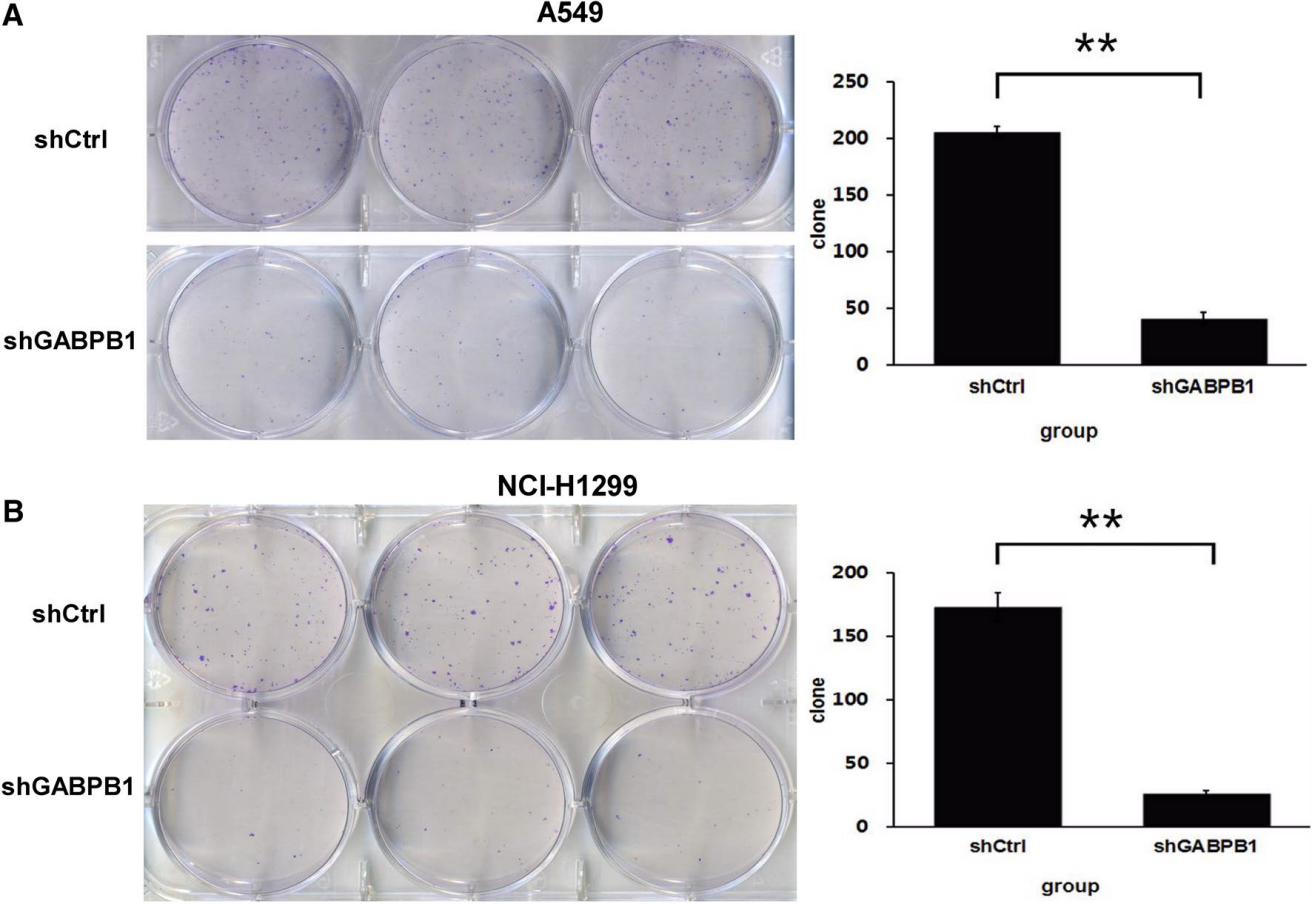
Effects of GABPB1 on lung cancer cell colony formation. The cell colony formation of A549 cells and H1299 cells were significantly inhibited after knockdown of GABPB1,compared shGABPB1 group with shCtrl group. **P < 0.01

To investigate the role of GABPB1 in the proliferation of two human NSCLC cell lines, we utilized a Celigo image cytometer and MTT assays. During the 5-day experimental period, the number of cells in the shCtrl group increased significantly compared with that in the shGABPB1 group for both the A549 and H1299 cell lines, especially after three days (P < 0.05). Viability assays showed that the number of viable A549 and H1299 cells was significantly lower in the shGABPB1 group than in the shCtrl group. Compared with that in the shCtrl group, the proliferation of the shGABPB1 group was lower (P < 0.05) (Fig. 5A and B). In A549 and H1299 cells, colony formation was obviously suppressed by the loss of GABPB1 in vitro (Fig. 6A and B). Overall, our findings suggest that GABPB1 plays an important role in the proliferation and colony formation capacity of NSCLC cells, as it significantly induces cell proliferation and colony formation.

### Knockdown of GABPB1 induced cell apoptosis in lung cancer

**Fig. 7:**
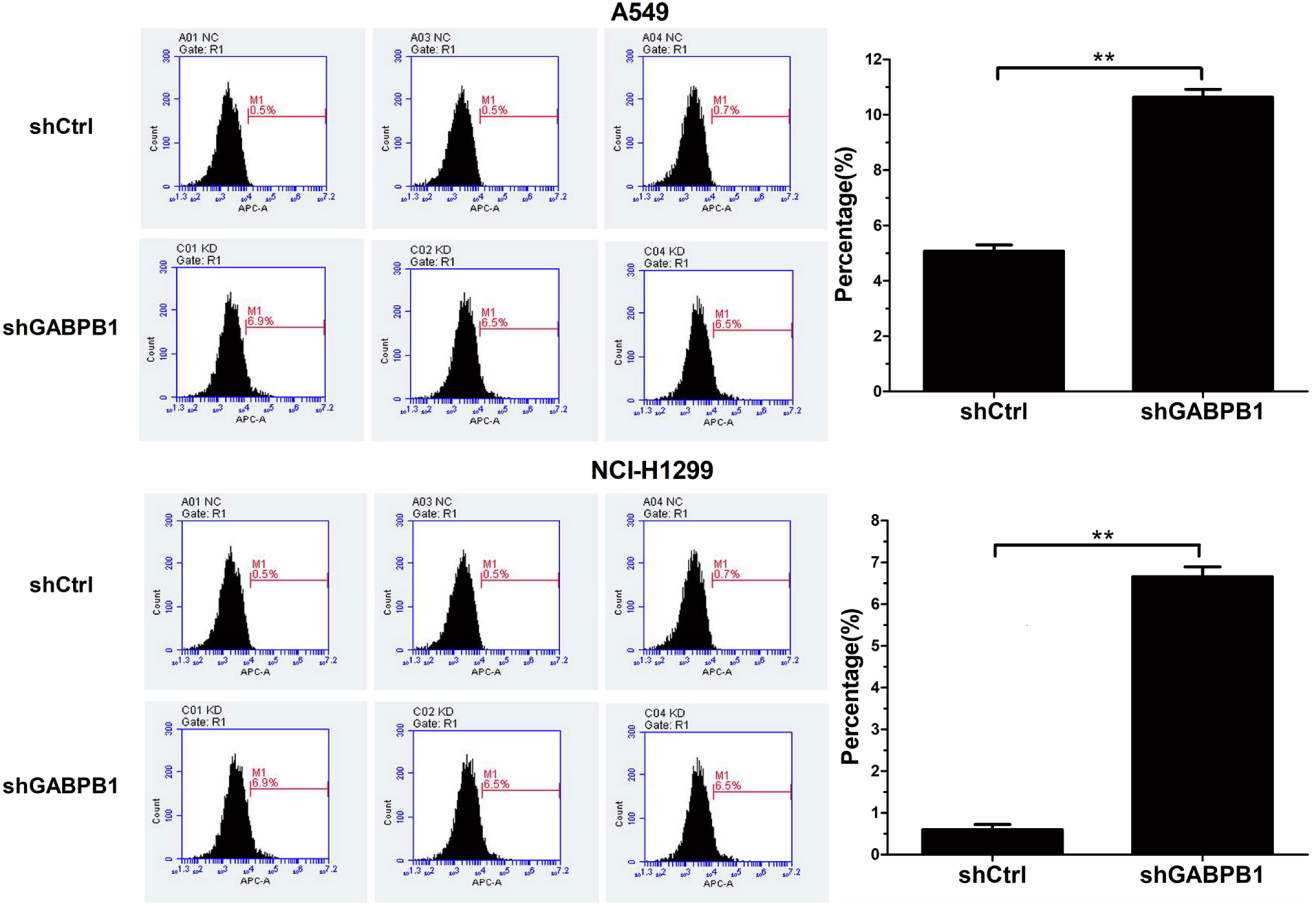
Effects of GABPB1 depletion on A549 cells and H1299 cells apoptosis. The apoptosis rate of A549 cells and H1299 cells increased in shGABPB1 group compared with shCtrl group. All experiments were performed independently at least three times. **P < 0.01

Apoptosis in lung cancer cells following GABPB1 knockdown was evaluated using flow cytometry. Three days after lentivirus infection, in both A549 and H1299 cells, the proportion of apoptotic cells was greater in the shGABPB1 group than in the shCtrl group (Fig. 7, P < 0.05). These findings suggest that downregulation of GABPB1 expression can induce apoptosis in NSCLC cells.

### High expression of GABPB1 was a poor prognostic factor for lung adenocarcinoma patients

**Fig.8:**
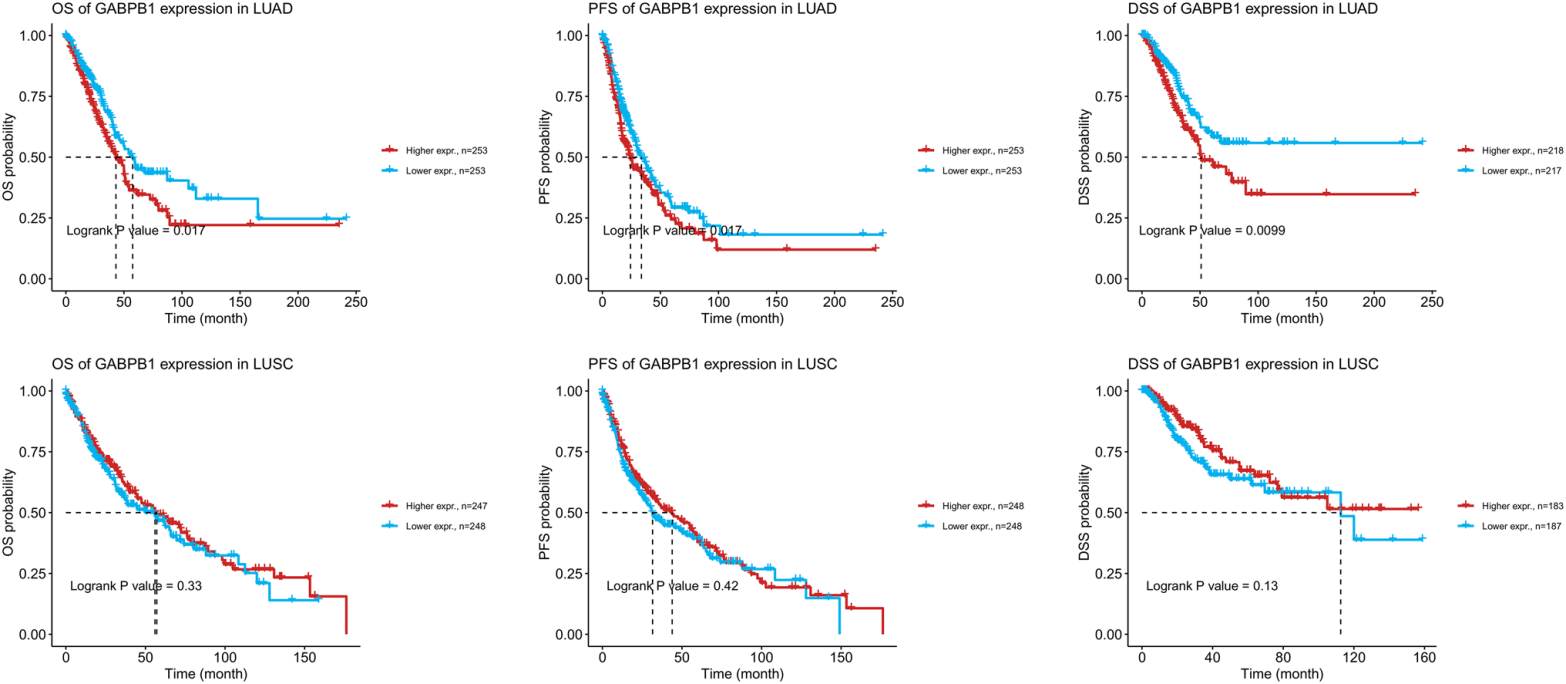
The correlation between the expression of GABPB1 and the prognosis of lung adenocarcinoma and lung squamous cell carcinoma patients

We further analysed the correlation between the expression of GABPB1 and patient prognosis. Observing OS, PFS and the DDS as indicators, our analysis revealed that high GABPB1 expression was indicative of poor prognosis in adenocarcinoma patients; however, our analysis did not reveal a similar conclusion for squamous cell carcinoma patients (Fig. 8).

### The expression and methylation of GABPB1 were related to the tumor immune microenvironment in NSCLC

**Fig.9:**
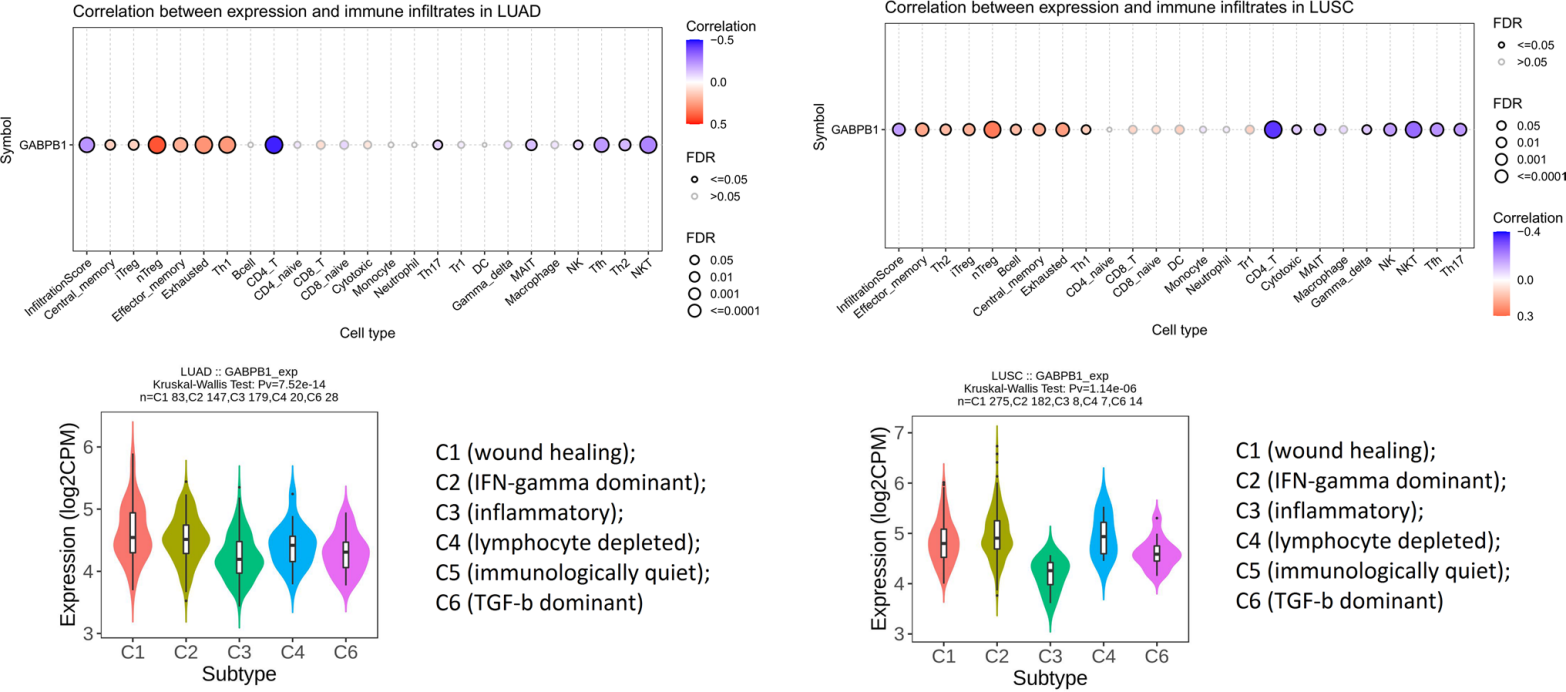
The expression of GABPB1 in LUAD and LUSC was related to immune infiltrates and immune subtypes

**Fig. 10:**
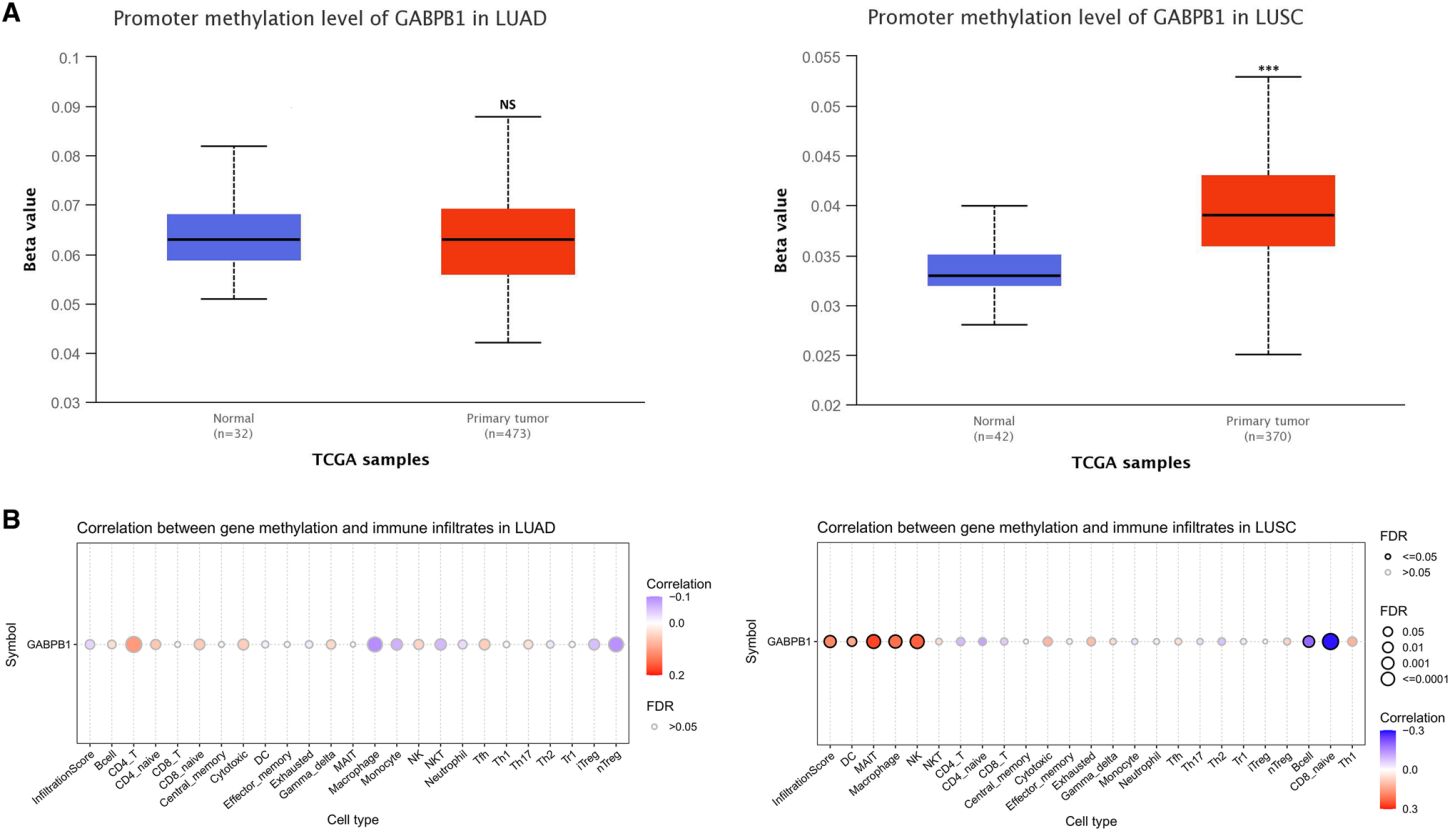
Methylation of GABPB1 changed its correlation with immune infiltrating cells. ***P < 0.001. **A** The expression of GABPB1 methylation in lung adenocarcinoma and squamous cell carcinoma. **B** The methylation of GABPB1 has been found to alter its correlation with tumor immune infiltrating cells

In NSCLC, the expression of GABPB1 was correlated with the infiltration of various tumor immune cells. Among these, the most significantly positive and negative correlations were observed for natural regulatory T cells (nTregs) and CD4 T lymphocytes, respectively. There were significant differences in the expression of this gene among the different immune subtypes. These findings showed similar trends in both lung adenocarcinoma and lung squamous cell carcinoma (Fig. 9). We also conducted an analysis of GABPB1 methylation in lung adenocarcinoma and lung squamous cell carcinoma using the TCGA database. However, the results showed a difference in GABPB1 methylation only between lung squamous cell carcinoma tissue and normal tissue (Fig. 10A). However, in both lung adenocarcinoma and lung squamous cell carcinoma, methylation alters the original association of GABPB1 with infiltrating tumor immune cells (Fig. 10B).

## Discussion

Despite extensive studies of emerging novel therapies, lung cancer remains the leading cause of cancer death both globally [17] and in China [18]. Most survival differences in lung cancer patients are caused by differences in the diagnostic stage. Due to the high proportion of patients with advanced-stage or metastatic disease at the time of diagnosis, the 5-year relative survival rate is low [19, 20]. Therefore, novel biomarkers that can predict treatment outcomes and prognosis are essential. Transcription factors, a diverse set of DNA-binding proteins that control genes, often show mutations, alterations in copy numbers, and translocations in cancer [21]. In lung cancer, these molecules can be sensitive diagnostic markers or promising prognostic markers and efficiently distinguish patients in early stages [22, 23]. GABP, which is distinguished by a conserved DNA-binding domain that can recognize nucleotide sequences with a GGAA/T core motif, is part of the E twenty-six (ETS) family of transcription factors [24]. GABP is composed of two subunits, with the transactivation beta subunit (GABPβ) regulating genes that are linked to a variety of basic cellular functions, such as cellular respiration in mitochondria [25], protein components of ribosomes [26], and cell cycle progression [27]. Numerous studies in the past have confirmed that the ETS transcription factor family participates in various physiological and pathological processes, including malignancy, during mammalian growth and development [28]. GABP may be associated with triggering the promoter in malignancies [29], but few studies have investigated the effects of GABPB1 in NSCLC. As a consequence, we have developed this study. At the start of the study, we examined the existing data on GABPB1 in the database, and the results revealed that GABPB1 was expressed in various human normal tissues and malignant tumors at both the protein and RNA levels. We tested paired lung cancer tissue and normal tissue after surgery and found that GABPB1 was expressed in multiple cells. Further analysis of the differential expression between tumor tissue and normal tissue showed that in the majority of tumor species, including NSCLC, there was a major discrepancy between paired malignant tumors and normal tissues. These findings provide a foundation for our future research.

Cell lines play an essential role in the study of oncogenesis and development and represent a suitable model for studying cell characteristics in terms of the proliferation index and malignancy [30]. Analysis of the database showed the expression of GABPB1 in the NSCLC cell line A549. We used this cell line as a control and found that there was relatively high expression of GABPB1 in all four NSCLC cell lines. Following our previous research, we analysed NSCLC A549 and H1299 cell lines, which are widely used in related research and exploration. RNAi technology can be used to specifically delete or inhibit the expression of specific genes. Researchers often use this method for gene silencing [31] to explore gene function and treat malignant tumors in vitro and in vivo with various tumor models [32]. shRNA, a major type of RNAi technology used to silence gene function, can be transfected or packaged into a recombinant virus and introduced into target cells [33]. Lentiviral vectors have several advantages, such as delivering siRNAs to nondividing cells, diminishing the risk of producing replication-competent viruses and preventing dysregulation of endogenous genes by promoters and viral enhancers [34]. Here, we successfully knocked down the expression of the GABPB1 gene in A549 and H1299 cell lines by lentivirus transfection. Furthermore, we examined the effect of knocking down GABPB1 expression on the regulation of cell proliferation and apoptosis. Several subsequent experiments confirmed that knocking down GABPB1 expression could reduce cell proliferation and increase cell apoptosis in both the A549 and H1299 cell lines. Our results confirmed that GABPB1 promotes tumorigenesis in vitro in NSCLC. Our experimental results in cell lines are similar to those of previous scholars. Chen et al. confirmed the increased expression of GABPB1 in renal cell carcinoma cell lines. Knockdown of GABPB1 in these cells significantly inhibited colony formation, and increased expression of GABPB1 was associated with poorer survival outcomes in patients with renal cancer [35]. This finding seems to confirm the cancer-promoting effect of GABPB1 in renal cell carcinoma. In patients with hepatocellular carcinoma (HCC), high expression of GABPB1 has been shown to correlate positively with poor prognosis. GABPB1 may be a new biomarker for HCC [12]. The high expression of GABPB1 in most malignant tumors and its research results in specific tumor types seem to confirm its cancer-promoting effect on malignant tumors. Scientists have studied the lncRNA GABPB1 intronic transcript in NSCLC and found that the expression of the GABPB1 intronic transcript was significantly downregulated in tumor samples from NSCLC patients and correlated negatively with tumor stage [36].

Previous studies have shown that the ETS transcription factor family is abnormally activated at all stages of tumorigenesis [37]. Following a large number of studies on ETS in patients with haematological malignancies [31], an increasing number of researchers are focusing on the relationship between ETS and solid tumors. The mechanisms controlled by ETS factors in solid tumors include cancer cell self-renewal and survival [38], DNA repair and genomic stability [39], alteration of the chromatin landscape [40], and modulation of metabolism [41]. Our in-depth database investigation revealed that GABPB1 expression is related to the immune subtypes of squamous cell carcinoma and adenocarcinoma of the lung. In addition, there are other findings in adenocarcinoma of the lung, such as differential expression of GABPB1 in different molecular subtypes and correlations between GABPB1 expression and activation-related signalling pathways, such as apoptosis, the cell cycle, and epithelial–mesenchymal transition (EMT). ETS factors are also related to the tumor microenvironment (TME), and some directly regulate extracellular matrix (ECM) remodelling and inflammation [31]; others contribute to the TME by regulating angiogenesis through the tumor-associated endothelium [42], and many ETS factors can be regulated in various ways [43]. Like that of other ETS family factors, the expression of GABPB1 was related to a variety of tumor immune infiltrating cells, and the expression of GABPB1 was consistently correlated with immune-infiltrating cells in both lung adenocarcinoma and squamous cell carcinoma. This molecule was positively associated with several immune regulatory cells and negatively correlated with immune-activating cells. Among the various immune subtypes, the expression of GABPB1 was relatively low in the infiltrating subtype, which was associated with a better immune response. These results suggest that GABPB1 may play a suppressive role in tumor immunity in NSCLC. Although there were no significant differences in the methylation of GABPB1 between lung adenocarcinoma and normal tissues, this was true for both lung adenocarcinoma and lung squamous cell carcinoma; methylation changed its correlation with immune infiltrating cells, which was more pronounced in squamous cell carcinoma. The correlation with immune infiltrating cells exhibiting different immunoregulatory effects showed the opposite direction, with attenuation of the tumor immunosuppressive effect exhibited by GABPB1. These changes may originate from the silencing effect of promoter methylation. Based on these results, further investigations of GABPB1 methylation could be performed to determine how to modify its immunosuppressive effects. Taken together, as a transcription factor, GABPB1 influences the development of lung cancer through various mechanisms, and the specific role of GABPB1 may vary in different types of cancer, which results in different prognostic impacts. In future research, we intend to further investigate the mechanisms underlying the impact of GAPB1 in adenocarcinoma and squamous cell carcinoma. By identifying the predominant population affected by GAPBP1-targeted therapy and determining the prognosis of lung cancer patients, we hope to find additional evidence to support the clinical application of GABPB1 in the diagnosis and treatment of lung cancer.

Our study attempted to observe the expression and explore the role of GABPB1 in NSCLC. The results showed that GABPB1 was overexpressed in human NSCLC tumor tissue and cell lines and that downregulation of GABPB1 inhibited cell proliferation and promoted cell apoptosis in vitro. The differential expression of GABPB1 was associated with clinical features and patient prognosis, and the impact of GABPB1 methylation on the tumor immune microenvironment may be a breakthrough point in this research. However, the role of GABPB1 in malignant tumors, especially lung cancer, has not been fully elucidated, and the relevant mechanisms also need to be further explored. However, further research is needed to provide additional evidence on the potential of GABPB1 as a biomarker or therapeutic target for clinical application.

## Data Availability

All relevant raw data, will be freely available to any researcher wishing to use them for non-commercial purposes, without breaching participant confidentiality.
